# Extraordinary Capacity of the Human Stomach

**Published:** 1812-06

**Authors:** 


*To the Editors of the Medical and Physical Journal.*

GENTLEMEN,

YOU will oblige a friend and constant reader, by inserting the following observations and case, if you deem them sufficiently interesting.

I am, your's, &c.

*London*, *March* 3, 1812. S. L.

The human stomach, from its fine texture, vascularity, and sensibility, is justly deemed a very delicate organ; yet it seems less liable to inflammation, than from its exquisite structure might be supposed. Not to mention the various alimentary matters with which it is, in many instances, daily gorged, and the ardent spirits with which it is stimulated, we have ample proofs of its powers of resisting the injurious effects of substances, yet more foreign from its nature, and more difficult of digestion, than Deady's double proof, or the richest compositions of Birch.* The stone-eaters, fire-eaters, and sword-swallowers, certainly surpass in their achievements the utmost efforts of the most determined gluttons. The swallowers of swords, I believe, are unknown in Europe, but exhibit their dangerous feats in the East Indies, to the great admiration of our countrymen. The fact has been attested by travellers of unquestionable authority, and has been scientifically investigated by a physician of the name of Johnson, who held the handle with one hand, whilst with the other he felt the point of the sword in the stomach of the operator. Upon withdrawing the instrument, some blood was observed upon it. The poor wretch had attained this art by accustoming himself to thrust down the gullet pieces of cane, whalebone, and other flexible substances, till he could bear iron itself.

The extraordinary case which occurred some years since at Guy's hospital, of the sailor who had been in the habit of swallowing large clasp-knives, would hardly have been credited had it been related by a traveller; or if it had not been confirmed by the authority of many respectable witnesses. The case, however, is not a solitary one, but, being so well attested, it inclines me not to reject, as absurd or impossible, similar instances, though not supported by such high authority. The following case is, perhaps, even more astonishing than the one to which I have just alluded, and would have obtained evidence from very few individuals, had not its possibility been established by that case. It is recorded in the "*Dictionaire des Merveilles de la Nature.*" Par M. A. I, S, D. Paris, 1781.

A galley-slave, a native of Nantes, entered the marine hospital at Brest the 5th of September, 1774. He complained of cough, pains in the stomach, and bowels; for which M. de Courcelles, physician for the quarter, prescribed some remedies which seemed to afford relief. On the 1st of October, M. Fournier, another physician to the hospital, began his quarter's attendance. The patient at this time complained of pains in the stomach and vomiting, which much fatigued him. Dr. Fournier not being able to ascertain the cause or nature of the complaints, directed some remedies

---

* Our friends in the country should be apprised that one of these characters is an eminent distiller, and the other a pastry-cook.—Ed.

which which he thought suitable to the case. The man died on the 10th day of the month, at two o'clock in the morning. On the following morning the body was opened. In the chest, on the left side, there was found an effusion of water, and suppuration had commenced in the lungs on the same side. Upon removing the teguments and muscles of the abdomen, the stomach was seen entirely displaced, occupying the left hypochondrium, and the lumbar and iliac region of the same side, and reaching down quite into the pelvis, close to the foramen ovale. Several hard bodies were felt in it. Dr. Fournier, conceiving the case worthy the attention of his colleagues, suspended further proceedings till the afternoon. In the mean time, the chest being open, he wished to trace the œsophagus its whole length. For this purpose he turned over the heart and lungs to the opposite side. In effecting this he ruptured the œsophagus, which occasioned him to perceive a piece of black wood, which reached from the beginning of the canal to the stomach itself. Notwithstanding this singular appearance, he waited till his colleagues arrived before he proceeded to gratify his curiosity.

At three o'clock in the afternoon, about fifty persons, including the physicians, surgeons, pupils, and attendants, were collected together. They first examined the position of the parts that are removed before we arrive at the opening of the stomach, which presented itself under the form of a long square, in which were distinguished four faces, each four inches in breadth, and containing the following substances, of which a detail was drawn up in presence of the spectators. The œsophagus, stomach, and most of the intestines, were tinged within with a black color, from the spot where the piece of wood before-mentioned was found, and which was a portion of a barrel hoop: all the substances also within the stomach were tinged with the same dark hue, and had a very fœtid odor, even after being washed several times.

*List of Articles found in the Stomach of the Man named Bazile.*

1. A portion of the hoop of a barrel, nineteen inches long, and one broad.

2. A piece of wood of the broom (genêt), six inches long, and half an inch in diameter.

3. A piece of the same, eight inches long, and half an inch in diameter.

4. A piece of the same, six inches long, diameter as before.

5. Ditto ditto, four inches long, ditto.

6. Ditto ditto, ditto, nearly cut in two in the middle.

7. A piece of wood of the oak, four inches and a half long, one inch and a half broad, and half an inch thick.

8. A portion

8. A portion of the same, four inches long, one inch broad, and eight lines in thickness.

9. A portion of the same, four inches long, half an inch broad, and four lines in thickness.

10. A portion of the same, four inches long, half an inch broad, and four lines in thickness.

11. A portion of the same, two inches long, one inch broad, and half an inch thick.

12. A portion of the same, four inches and a half long, and four lines broad on each of its faces.

13. A portion of the same, four inches long, of a triangular form, and with a surface of four lines.

14. A portion of the same, four inches long, and four lines in diameter.

15. A portion of the same, five inches long, half an inch broad, and two lines thick.

16. A portion of the same, five inches long, four lines broad, and two lines thick.

17. A portion of the same, of irregular form, three inches long, and three lines in thickness.

18. A portion of the same, three inches long, half an inch broad, and three lines in thickness.

19. A piece of hoop of a barrel, five inches long, one inch broad, and two lines in thickness.

20. A piece of deal, four inches long, one inch broad, and five lines thick.

21. A piece of the same, four inches long, and four lines in diameter.

22. A piece of the same, two inches and a half long, and one inch broad.

23. A piece of the same, three inches long, half an inch thick, and of irregular form.

24. A piece of the same, two inches and a half long, and four lines thick.

25. A portion of bark from a hoop, three inches and a half long, and one inch broad, being part of the large piece detached from the superior portion which was in the œsophagus, and which had descended into the stomach.

26. A lump of wood, an inch long, and an inch in diameter.

27. A wooden spoon, five inches long, and an inch and a half broad.

28. A tube of a tin funnel, three inches and a half long, an inch in diameter in the upper part, and half an inch on the inferior part.

29. Another piece of a funnel, two inches and a half long, and half an inch in diameter.

30. The handle of a tin spoon, four inches and a half long.

31. An entire tin spoon, seven inches long, the bowl of the spoon bent back.

32. Another spoon of the same metal, three inches long.

33. Another ditto, two inches and a half long.

34. A piece of iron, two inches and a half long, half an inch broad, and four lines in thickness, weighing one ounce, four drachms and a half.

35. A bowl of a tobacco pipe, with part of the tube, three inches long.

36. A nail half polished, without the point, with its head, two inches long.

37. A sharp-pointed nail, an inch and a half long.

38. A piece of a tin spoon flattened, an inch long, half an inch broad.

39. Three pieces of metal buckle, of irregular form, each about half an inch long.

40. Five plumb-stones.

41. A small piece of horn.

42. Two pieces of white glass, the largest an inch and four lines in length, half an inch in breadth, and of irregular form.

43. Two pieces of leather, the largest three inches long, an inch broad, and of irregular form; the other an inch and four lines long, and half an inch broad.

44. A knife with its blade, wood handle, bent, three inches and a half long, and an inch in its greatest breadth.

The total of these several articles was fifty-two pieces, weighing all together one pound, ten ounces, and four drachms.

M. Fournier, who published the particulars of this case, observes, "we cannot but regret the silence of this unfortunate patient respecting the cause of his complaints. If it had been possible for me to have suspected it, I might have asked him questions which, perhaps, would have led to some explanation of so extraordinary a phenomenon. Since his death I have made every imaginable inquiry into his character, temperament, and habits. All the information I obtained in consequence was, that he was naturally hypochondriacal, and rather weak in intellect. He had been a soldier in the marines for thirteen years, and was discharged from the service from his head being deranged. Among other things, his comrades often persuaded him that he was very ill, to which he answered he believed he was, and took to his bed. At that time he had a great appetite, and ate much. Dismissed from the Royal Corps, he returned to Nantes, where, after some time, he was condemned to the galleys. One of his companions, who was subjected to the same punishment, and who was with him in prison, assured me that he has often seen him scrape the lime and mortar which covered the walls of the prison, and put a large quantity of them in his soup, saying that it strengthened him and gave him courage. He also added, that sometimes he had a ravenous appetite, which was attended with copious salivation; he he would then eat as much as would suffice four men. But, if he could not obtain a sufficient supply of food, which often happened, for he was passionately fond of tobacco, and sold his rations to procure it, he then would swallow small stones, buttons, leather gaiters, and other substances.

"Having also interrogated the men who were on the same bench with him where he was confined, they declared that, two days before his entrance into the hospital, they had seen him swallow two pieces of wood four or five inches long. But, after all the inquiries I was able to make, I could not ascertain when he had swallowed the enormous piece of hoop of nineteen inches."

Since his admission in the hospital, his drink passed regularly, but he took very little solid food.

It appeared, from considering all the circumstances of the case,

1. That the foreign bodies could not have been put into the man's stomach after death, as by some individuals it was supposed, because, 1st, The great derangement of the stomach could only have taken place gradually, and was probably occasioned by the weight of the substances it contained. 2dly, Strong adhesion had taken place between it and the edge of the foramen ovale, where even gangrene was occasioned by the pressure and rubbing of the large piece of hoop. 3dly, The black color of all the pieces, which appeared as if macerated, exhaled a very fœtid odor, and had tinged the intestines with the same color. 4thly, His sufferings, of which he did not complain till the last days, though his neighbours affirmed that he had suffered a considerable time, from the colic pains which troubled him during his stay in the hospital; the little solid food which he took; and lastly his own evidence, for one of the sisters remembered that he said, *he had a thousand devilish things in his body which would kill him*, to which she then paid little attention, considering him mad or foolish.

2. It is very probable, and even averred, that his mind was deranged, and that the digestive juices, vitiated by whatever cause, occasioned in him at intervals that raging hunger, which not having wherewithal to satisfy, he swallowed any thing that came in his way.

3. It appeared that he had acquired this habit gradually, and that at first he was accustomed to swallow small substances, which had passed in the usual way; and that he was unfortunately persuaded that the other bodies which he swallowed latterly, would also pass as readily, considering the intestinal canal as a straight tube, in which what entered from above, must necessarily pass downwards without any obstruction. If it is easy to demonstrate that his sufferings were entirely the result of what was found after his death; if it is equally easy to convince ourselves of the truth of the fact by authentic testimonies and attestations; it is not equally possible to imagine and explain why he did not suffer more acute, severe, and characteristic, symptoms, and especially how he could make a piece of wood nineteen inches long pass the pharynx and œsophagus without rupturing the parts, or causing him to be choaked.

